# Dynamic Folding Pathway Models of the Trp-Cage Protein

**DOI:** 10.1155/2013/973867

**Published:** 2013-06-24

**Authors:** In-Ho Lee, Seung-Yeon Kim

**Affiliations:** ^1^Korea Research Institute of Standards and Science, Daejon 305-600, Republic of Korea; ^2^School of Liberal Arts and Sciences, Korea National University of Transportation, Chungju 380-702, Republic of Korea; ^3^Department of Physics and Astronomy, University of South Carolina, Columbia, SC 29208, USA

## Abstract

Using action-derived molecular dynamics (ADMD), we study the dynamic folding pathway models of the Trp-cage protein by providing its sequential conformational changes from its initial disordered structure to the final native structure at atomic details. We find that the numbers of native contacts and native hydrogen bonds are highly correlated, implying that the native structure of Trp-cage is achieved through the concurrent formations of native contacts and native hydrogen bonds. In early stage, an unfolded state appears with partially formed native contacts (*~*40%) and native hydrogen bonds (*~*30%). Afterward, the folding is initiated by the contact of the side chain of Tyr3 with that of Trp6, together with the formation of the N-terminal **α**-helix. Then, the C-terminal polyproline structure docks onto the Trp6 and Tyr3 rings, resulting in the formations of the hydrophobic core of Trp-cage and its near-native state. Finally, the slow adjustment processes of the near-native states into the native structure are dominant in later stage. The ADMD results are in agreement with those of the experimental folding studies on Trp-cage and consistent with most of other computational studies.

## 1. Introduction

The understanding of the folding dynamics of a protein from its one-dimensional amino-acid sequence into the three-dimensional native structure is a long-standing challenge in modern science. Although the extensive studies on protein folding dynamics have been performed, many aspects of protein folding dynamics are poorly understood. Due to the difficulties in the understanding of folding dynamics for large proteins, fragments of proteins (e.g., *α*-helix and *β*-hairpin) and small proteins have been mainly used to investigate protein folding dynamics. Recently, the 20-residue Trp-cage protein [1] with a fast folding rate [2] has attracted many researchers, both experimentalists [1–11] and theoreticians [12–24], in the protein-folding research community.

The Trp-cage protein has the amino-acid sequence of NLYIQ WLKDG GPSSG RPPPS (PDB code: 1L2Y). The PDB structure of Trp-cage contains the *α*-helix in residues from 2 to 8, the 3_10_-helix in residues from 11 to 14, and the C-terminal polyproline II structure. In the hydrophobic core of the Trp-cage, several hydrophobic residues (e.g., tyrosine and proline residues) surround the central Trp6 residue. Also, the salt bridge between Asp9 and Arg16 is important for the Trp-cage stability.

In this work, action-derived molecular dynamics (ADMD) [25–27] and parallel computation are used to investigate the folding pathway models of the Trp-cage protein into the native structure at all-atom resolution. The ADMD method is useful for the study of rare events, especially for protein folding study. The ADMD method has been successfully applied for searching the dynamic folding pathway models of the fragments *α*-helix (acetyl-(Ala)_10_-N-methyl amide) and *β*-hairpin (residues 41–56 of protein G) [28], the villin headpiece subdomain (HP-36) structure [29], and the miniprotein FSD-1 [30]. In the previous applications, the obtained dynamic pathway models for *α*-helix, *β*-hairpin, HP-36, and FSD-1 have been consistent with experimental data, demonstrating that much insights can be obtained through ADMD studies.

In ADMD, by applying the least action principle, the initial value problem is converted into the boundary value problem for obtaining classical Newtonian trajectories. We directly search the protein-folding pathway for the given initial and final conformations. The goal of this study is to investigate the dynamic folding pathway models of the Trp-cage protein by providing its sequential conformational changes from its initial disordered structure to the final native structure, at atomic details. The time interval between successive conformational changes is set to be short enough to describe the folding event in structural continuity, but long enough so that not-so-important fast vibrational modes are properly averaged out.

## 2. Methods

In ADMD, by applying the least action principle, the Newtonian dynamics formulation is now transformed into a boundary value problem to generate classical low-potential-energy trajectories bridging two given structures. We relate trajectories with low-potential-energy barriers as probable transition pathways. An appealing feature of ADMD is that its trajectory globally follows a Newtonian trajectory according to the equations of motion [25–27]. In ADMD simulations for the Trp-cage protein, the whole atomic trajectory is discretized in *P* = 2000 steps. The total simulation time is *τ* = *P*Δ. The path **q**(*t*) is represented by the initial state **q**_0_, the final state **q**_*P*_, and the states (**q**_1_, **q**_2_,…, **q**_*P*−1_) at the intermediate time levels *t*_1_, *t*_2_,…, *t*_*P*−1_. The path {**q**_*j*_} is a collection of sequential structural frames with the fixed initial **q**_0_ and final **q**_*P*_. Then the classical action can be written as

(1) $$\begin{array}{c} S=\sum_{j=0}^{P-1}L_{j}\left(\left\{q_{j}\right\}\right)\Delta , \end{array}$$

where the discretized Lagrangian of the *j*th temporal frame is defined as

(2) $$\begin{array}{c} L_{j}=\sum_{I=1}^{N}\frac{m_{I}}{2\Delta^{2}}{\left(q_{I,j}-q_{I,j+1}\right)}^{2}-V\left(\left\{q_{j}\right\}\right). \end{array}$$

Here, the first term is the kinetic energy and *V* is the potential energy. *N* is the total number of atoms, *m*_*I*_ is the mass of the *I*th atom, and **q**_*I*,*j*_ is the position vector of the *I*th atom at the *j*th frame.

The stationarity condition *δS* = 0 leads to a set of linear equations. However, discretized pathways generated from the minimization of (1) do not satisfy total energy conservation as discussed in the work of Passerone and Parrinello [25]. That is, accurate Verlet trajectories are not guaranteed since the action of (1) is not bounded. Passerone and Parrinello [25] suggested adding a constraint term to (1) to ensure the total energy conservation from pathways. The modified action (the so-called Passerone-Parrinello action) is defined by

(3) $$\begin{array}{c} \Theta \left(\left\{q_{j}\right\};E\right)=S+\mu_{E}\sum_{j=0}^{P-1}{\left(E_{j}-E\right)}^{2}, \end{array}$$

where *E* is the target total energy value to impose on the system, *μ*_*E*_ is an arbitrary large constant, and *E*_*j*_ is the total energy at the *j*th frame defined as

(4) $$\begin{array}{c} E_{j}=\sum_{I=1}^{N}\frac{m_{I}}{2\Delta^{2}}{\left(q_{I,j}-q_{I,j+1}\right)}^{2}+V\left(\left\{q_{j}\right\}\right). \end{array}$$

The quality of pathways can be improved by adding the following dynamic restraint [26]:

(5) $$\begin{array}{c} R=\mu_{K}\sum_{I=1}^{N}{\left(\langle K_{I}\rangle -\frac{3k_{B}T}{2}\right)}^{2}, \end{array}$$

to the Passerone-Parrinello action, where *μ*_*K*_ is an arbitrary large constant, 〈*K*_*I*_〉 is the average kinetic energy of the *I*th atom along the trajectory, *k*_*B*_ is the Boltzmann constant, and fictitious temperature *T* controls the kinetic energy of the system. Consequently, we optimize the extended action

(6) $$\begin{array}{c} \Phi \left(\left\{q_{j}\right\};E,T\right)=\Theta +R \end{array}$$

to obtain protein-folding pathways at the all-atom resolution.

In this work, we have optimized the extended action, (6), to generate ADMD pathways with 3*N*(*P* − 1) = 3 × 304 × 1999 = 1823088 degrees of freedom (the number of atoms *N* = 304 for Trp-cage). All atoms are treated as point particles with atomic masses according to their atom types (H, C, N, and O). It should be noted that no artificial constraints are imposed on the covalent bond lengths and angles other than that they are subject to the force field used. We used the AMBER all-atom force field [12, 15–18, 20–22, 24, 31] and the GB/SA solvation potential [12, 13, 15–18, 21, 32] to evaluate the interatomic potential energies of the protein structures. Folding simulations are performed without the help of any constraints on molecular structural change.

To start ADMD simulations, the initial and final coordinates of the atoms should be provided. In this work, the final conformation is obtained, after a local energy minimization, from the PDB structure. The choice for the initial conformation is less obvious, and we used a local-energy-minimized structure (obtained through a few-minute run of Newton minimization in the TINKER package [33] on a Linux PC), starting from the fully extended conformation. The initial conformation has the radius of gyration *R*_*g*_ = 10.2 Å (much larger than the experimental value [5], 8.0 ± 0.2 Å, of the unfolded state), a large value (8.4 Å) of the root-mean-square deviation (RMSD) from the final conformation, no native contact, no hydrogen bond, no contact between the side chain of Trp6 and the side chains of the other residues, and no salt bridge. That is, the initial conformation is a completely disordered state. The potential-energy difference between the initial and final conformations is measured to be 46.61 kcal/mol. At the beginning of each ADMD simulation, a set of random numbers is generated to construct a trial atomic trajectory for each atom, connecting the initial and the final conformations provided.

To estimate the value of the optimal target energy *E*, several preliminary ADMD runs are carried out. The first preliminary run is executed with an overestimated value of *E*. After an ADMD solution is obtained with *E*, successive runs are tried with lower (typically by 1~2 kcal/mol) values of *E*. For each successive run, the previous ADMD solution is used as the starting trajectory in an iterative way. The final value of *E* is set as the smallest, which provides a solution satisfying the total-energy conservation along the folding trajectory. It should be noted that if the value of *E* is set too low, ADMD trajectories fail to satisfy the total-energy conservation. Also, it should be noted that *T* used in this work does not correspond to the physical temperature. *T* is only a parameter introduced to improve the quality of pathway by reducing the value of Onsager-Machlup action [25–27]. A smaller value of Onsager-Machlup action corresponds to a more Verlet-like trajectory.

For the rigorous minimization of the extended action defined in (6), one should consider applying a global optimization method such as simulated annealing. However, since the execution of even a local minimization takes a significant amount of computational resources, we decided to perform separate local minimizations. For the local minimization, a multigrid method [34] is used where the number of conformations (*P*), initially as small as 20, continues to grow to reach 2000 at the final stage. For a given *P* we used the quasi-Newton relaxation method, L-BFGS routine [35] with its default stop condition.

The trajectory for each atom can be represented by sine expansion [27]:

(7) $$\begin{array}{c} q_{j}=q_{0}+\left(q_{P}-q_{0}\right)\frac{j\Delta }{\tau }+\sum_{k=1}^{P-1}a_{k}\sin\left(\frac{k\pi j\Delta }{\tau }\right). \end{array}$$

Now, the positions of each atom along the trajectory are represented by 3(*P* − 1){**a**_*k*_} variables in (7). Finally, (6) is minimized with respect to 3*N*(*P* − 1) = 1823088-independent variables. It should be noted that {**a**_*k*_} provides a natural way to interpolate a pathway, which works well with the multigrid (from *P* = 20 to *P* = 2000) approach used in this work.

## 3. Results

We have carried out twenty independent ADMD calculations where initial pathways are prepared in a random fashion. An initial pathway constitutes a set of successive conformations prepared in real space, and the difference between two successive conformations is set by using random numbers. When analyzing the ADMD simulation data, in order to eliminate possible artifacts arising from the choice of an initial pathway, we have extracted common folding features representing the twenty final pathways.

The purpose of the ADMD simulation is to find pathways bridging two given states with low potential-energy profile while satisfying the equations of motion. Considering all pathways starting from the given initial structure and arriving at the given final structure following the Newtonian equations of motion, we aim to identify pathways with low potential energy barriers. The potential energy barrier is defined as the potential energy difference between the highest potential energy state and the initial state. Since the entire pathway ensemble satisfying the boundary conditions could not be considered, we hope that a total of twenty low potential energy pathways performed in this work would provide meaningful characteristics of folding mechanism. For each ADMD trajectory, sequential folding event is analyzed in terms of various quantities including the secondary structure element and the overall degree of collapse. Indeed, although details of all twenty ADMD simulations were different from each other, we were able to extract common features of folding. This demonstrates that even a small protein-like Trp-cage can exhibit a specific folding sequence governed by the energetics of the conformational space.

Each of ADMD simulations produced a low potential-energy pathway. Initial pathways were prepared in a random fashion, producing variation in pathways. However, these twenty pathways show similar potential-energy fluctuation along their trajectories, and overall folding features independent of initial randomness are considered. We have selected the lowest potential-energy pathway out of twenty to illustrate the features.

**Figure 1:**
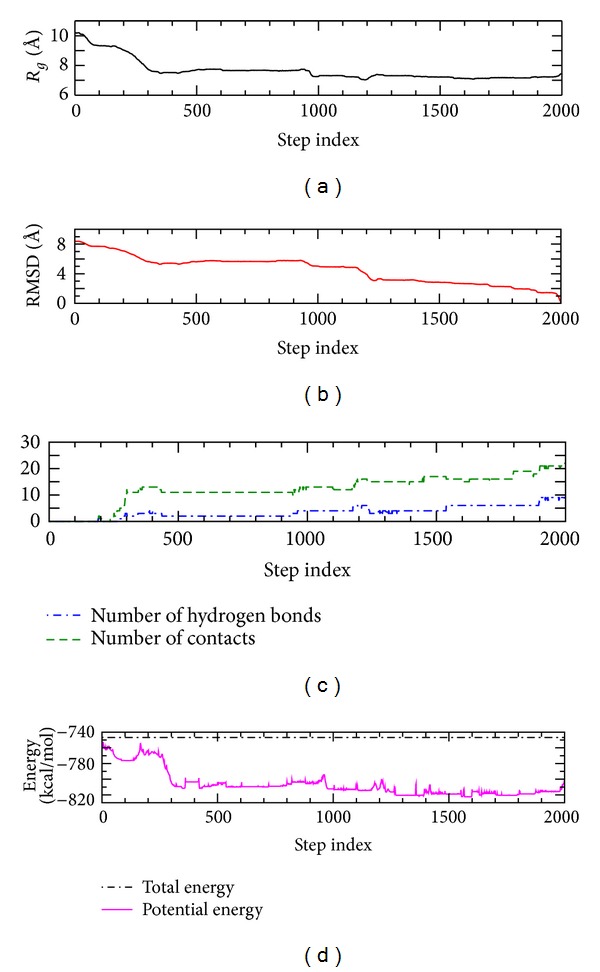
The radius of gyration (*R*_*g*_), the root-mean-square deviation (RMSD) from the final native structure, the numbers of native contacts and hydrogen bonds, the total energy, and the potential energy for Trp-cage folding, as a function of the time step index. The total energy is well conserved for the whole-time steps.

In the analysis of ADMD simulations, the folding sequence is investigated for the formation of secondary and tertiary structures, the overall degree of collapse, and the packing of the Trp6 side chain, and compared with other studies. The overall feature of folding dynamics is shown with the set of variables (such as the radius of gyration (*R*_*g*_), RMSD from the final native structure, and potential energy) as a function of the time step index *j* (see Figure 1). The variations in the numbers of native contacts and hydrogen bonds are also shown in the figure.

**Figure 2:**
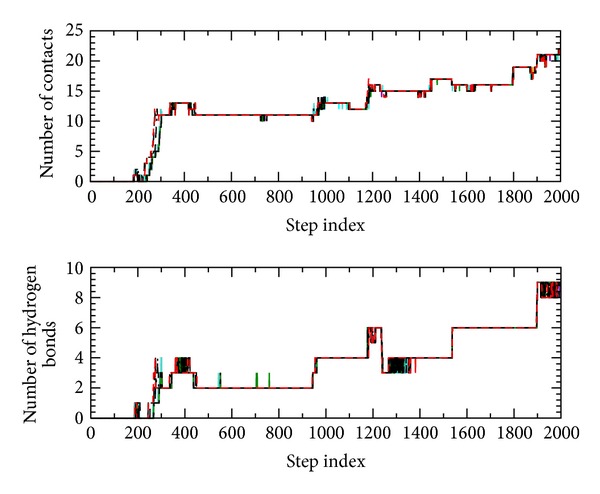
The numbers of native contacts and hydrogen bonds, as a function of the time step index, for all twenty independent ADMD pathways of Trp-cage folding.

Figure 2 shows the numbers of native contacts and hydrogen bonds, as a function of the time step index, for all twenty independent ADMD pathways of Trp-cage folding. As shown in the figure, the overall behavior is similar along these twenty folding pathways, and differences among the pathways are not so large. Also, other quantities follow the same trend for these twenty pathways.

We calculated the numbers of native contacts (responsible for the formation of tertiary structure) and native hydrogen bonds (responsible for secondary structure) to quantify the degree of folding process. A native contact is defined to exist between two residues (separated by more than two residues in sequence) if their native C^^*α*^^-C^^*α*^^ distance is less than 6.5 Å. A backbone hydrogen bond is defined to exist between a carbonyl-oxygen and an amide-hydrogen if they are separated by less than 2.5 Å, and the virtual bond angle between three atoms (oxygen, nitrogen, and amide-hydrogen) is greater than 135°.

As shown in Figure 1, the structural variables, RMSD and *R*_*g*_, are correlated along the folding pathway since the linear correlation coefficient *r* = 0.79 for the whole 2001 conformations. The linear correlation coefficient is *r* = 0.99 for the first 1000 steps, indicating a strong correlation between RMSD and *R*_*g*_ at the early stage of protein-folding process. Similarly, there is a clear correlation between potential energy and RMSD, consistent with other computational result [12]. A notable pairwise relatedness between potential energy and RMSD (*R*_*g*_) is present. Also, the numbers of native hydrogen bonds and native contacts are highly correlated, as linear correlation coefficient *r* = 0.89, along the folding pathway. Therefore, the native structure of Trp-cage is achieved through the concurrent formations of native contacts and native hydrogen bonds.

**Figure 3:**
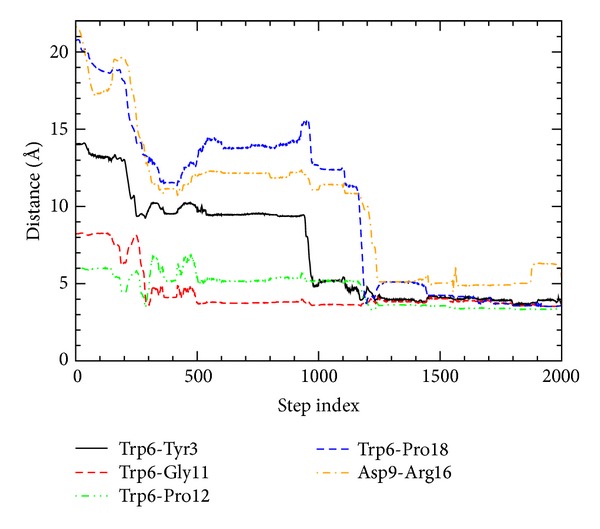
The distance between the side chains of Trp6 and Tyr3 and the distances for Gly11, Pro12, and Pro18 from the side chain of Trp6, as a function of the time step index. In addition, the distance between the side chains of Asp9 and Arg16 (which form the salt bridge in the native structure) is shown.

In addition, to investigate the packing process of the tryptophan residue as a function of the time step index *j*, we have measured the distance between the side chains of Trp6 and Tyr3 and the distances for Gly11, Pro12, and Pro18 from the side chain of Trp6, as shown in Figure 3. Also, the figure shows the distance between the side chains of Asp9 and Arg16 which form the important salt bridge in the native structure.

As a further analysis of the Trp-cage folding processes, the method of principal component analysis is also applied. This method extracts the essential motions in the protein-folding events through the ADMD simulation. The results of principal component analysis are shown in Figures 5, 6, and 7.

### 3.1. Early Stage

In early stage, a local potential-energy increment is present around the time step index *j* ~ 170, as shown in Figure 1. This implies that there are possible energy hills. Around the possible energy hill regime, the protein structure does not have any sign of formation for native contacts and native hydrogen bonds. Thus, the protein conformations found at the early step indices (*j* < 170) in the present ADMD simulation could be categorized as completely disordered states, in general.

For the time step indices 170 ≤ *j* ≤ 300, Figure 1 shows the sudden simultaneous increases in the numbers of native contacts and hydrogen bonds and the sudden decrease of potential energy, together with the simultaneous decreases of RMSD and *R*_*g*_ (implying chain compaction). Within the step indices 237 ≤ *j* ≤ 300, the sequence of the native contact formations is as follows: (3,6) → (5,8) → (7,10) → (7,11) → (9,14) → (11,14) → (6,11) → (12,16) → (12,15), where (3,6) represents the native contact between the third and sixth residues. The helical contact between Tyr3 and Trp6 initiates the contact formations, in good agreement with other folding study of Trp-cage [16]. The (*i*, *i* + 3)-type native contacts (3,6) and (5,8) appear in the N-terminal fragment, which will become the *α*-helix in the native structure, and the contacts (11,14) and (12,15) are related to the 3_10_-helix in the middle fragment. It seems that the formation of the (*i*, *i* + 3)-type native contacts precedes that of the (*i*, *i* + 4)-type native contacts in the *α*-helix [28].

**Figure 4:**
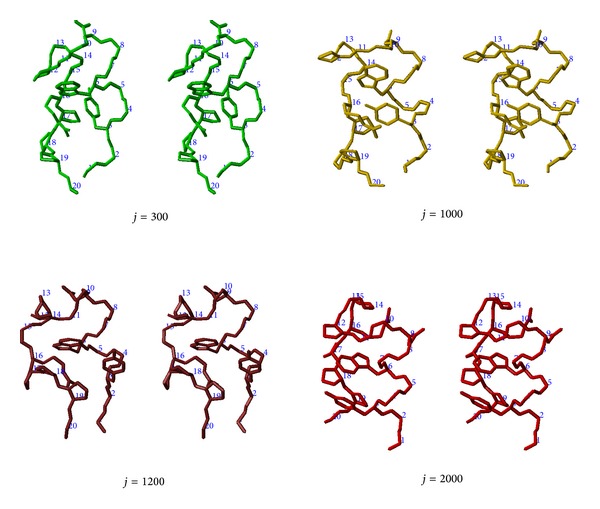
The stereographic view for the conformations at *j* = 300, 1000, 1200, and 2000. The side chains for Tyr3, Trp6, Asp9, Pro12, Arg16, Pro17, Pro18, and Pro19 are shown. The final conformation at *j* = 2000 is the native structure.

At the step index *j* = 300, an unfolded state with partially formed native contacts (~40%) and native hydrogen bonds (~30%) appears (see also Figure 4). It includes two partially formed 3_10_ helices in the N-terminal and middle fragments. Also, other computational studies on Trp-cage reported the partial formations of the helical elements in the unfolded state [15–17, 20, 22, 23]. Similarly, experimental studies showed the existence of the helical elements in the unfolded state of Trp-cage [3, 11], and another experimental study emphasized the importance of preformed structure in the unfolded state for its fast folding [4]. The conformation at *j* = 300 shows the RMSD value of 5.6 Å, quite different from the native structure. Its radius of gyration is *R*_*g*_ = 7.8 Å, in agreement with the experimental value [5], 8.0 ± 0.2 Å, of the unfolded state. A recent computational study using replica-exchange molecular dynamics [24] has also reported the unfolded state with RMSD ~5.2 Å and *R*_*g*_ ~ 8 Å, close to our values.

As shown in Figure 3, the distances between the side chains of Trp6 and Tyr3, between Trp6 and Pro18, and between Asp9 and Arg16 (salt bridge) decrease simultaneously during 170 ≤ *j* ≤ 300 but are quite far from the native values. No salt-bridge formation in the unfolded state is consistent with most of other experimental [5] and computational [13, 16, 17, 20] studies. In contrast, using replica-exchange molecular dynamics and the OPLSAA force field, a computational study reported the salt-bridge formation in the unfolded state with *R*_*g*_ ≈ 9.4 Å and about 42% of native contacts [14]. Also, using replica-exchange molecular dynamics, transition path sampling, and the OPLSAA force field, another computational study reported the presence of the salt bridge in the unfolded states [19]. However, using replica-exchange molecular dynamics and the OPLSAA force field again, a recent computational study reported no salt-bridge formation in the initial state (with *R*_*g*_ ≈ 7.6 Å and about 17% of native contacts), the intermediate state (with *R*_*g*_ ≈ 7.2 Å and about 42% of native contacts), and the transition state (with *R*_*g*_ = 7.3 Å and about 55% of native contacts) [23].

On the other hand, the distances between Trp6 and Gly11 and between Trp6 and Pro12 become close to the native value at *j* ~ 300, as shown in Figure 3. Therefore, in the unfolded state, the side chain of Trp6 is in contact with the residues (Gly11 and Pro12) of the middle 3_10_ helix, consistent with an experimental study [3]. According to a recent experimental study on Trp-cage [5], the side-chain contact of Trp6 with Pro12 exists in its unfolded state, and the close interaction between Trp6 and Pro12 contributes to its fast folding.

For the time step indices 300 ≤ *j* ≤ 900, there is no remarkable change for *R*_*g*_, RMSD, and the numbers of native contacts and hydrogen bonds, as shown in Figure 1. During this period, the unfolded state is quite stable with the average values of *R*_*g*_ = 7.65 ± 0.07 Å, RMSD = 5.62 ± 0.13 Å, the number of native contacts = 51 ± 3%, and the number of native hydrogen bonds = 24 ± 4%. At the step index *j* = 900, eleven native contacts are formed such as (3,6), (4,7), (5,8), (6,9), (6,10), (6,11), (7,10), (9,14), (11,14), (12,15), and (12,16). Compared to the conformation at *j* = 300, the native contact (7,11) disappears but the new native contacts (4,7), (6,9), and (6,10) appear. The (*i*, *i* + 3)-type native contacts (3,6), (4,7), (5,8), and (6,9) are still dominant in the N-terminal region. According to Figure 3, the distances from the side chain of Trp6 and the distance of the salt bridge show some oscillations during 300 < *j* < 500, and they vary little during 500 < *j* < 900.

### 3.2. Folding into the Native Structure

Around *j* ~ 950, as shown in Figure 1, *R*_*g*_ and RMSD decrease slightly and the numbers of native contacts and hydrogen bonds increase slightly, together with a slight change of the potential energy. The most remarkable change around *j* ~ 950 is the sharp decrease of the distance between the side chains of Trp6 and Tyr3, as shown in Figure 3. The distance decreases from 9.4 Å at *j* = 940 to 4.9 Å at *j* = 980. Finally, the side chain of Tyr3 is in contact with that of Trp6. In particular, the hydrophobic stacking of the aromatic rings of Tyr3 and Trp6 is identified as the key interaction in the Trp-cage folding processes from a recent experiment [8].

At the same time (940 ≤ *j* ≤ 980), the radius of gyration decreases from 7.7 Å to 7.3 Å and RMSD from 5.7 Å to 5.1 Å. During the same period, the numbers of native contacts and hydrogen bonds increase. For example, at *j* = 971, about 60% of native contacts and about 45% of native hydrogen bonds are formed. More importantly, at *j* = 971, two (*i*, *i* + 4)-type pairs (3,7) and (4,8) appear for both native contacts and native hydrogen bonds, indicating the first formation of the N-terminal *α*-helix. Figure 4 shows the conformation at *j* = 1000, resulting from the changes around *j* ~ 950.

After *j* = 1000, the most remarkable event is the dramatic decrease of the distance between Pro18 and the side chain of Trp6, for the time step indices 1150 ≤ *j* ≤ 1200, as shown in Figure 3. The distance decreases from 11.3 Å at *j* = 1150 to 3.9 Å at *j* = 1200. That is, the C-terminal polyproline II structure docks onto the Trp6 and Tyr3 rings of the partially formed N-terminal *α*-helix, in agreement with a recent experimental study [9]. Finally, the side chain of Tyr3, the 3_10_ helix in the middle region, and the C-terminal polyproline II structure surrounds the side chain of Trp6, indicating the formation of the hydrophobic core of Trp-cage. The conformation at *j* = 1200 is shown in Figure 4. For the step indices 1150 ≤ *j* ≤ 1200, the radius of gyration decreases slightly from 7.2 Å to 7.1 Å and RMSD from 4.9 Å to 3.9 Å, as shown in Figure 1. Also, the numbers of native contacts and hydrogen bonds increase at the same time. For example, at *j* = 1190, 73% of native contacts and 67% of native hydrogen bonds are formed. In particular, the long-range native contact (3,19) between Tyr3 and Pro19 and the proline-proline native contact (12,17) between Pro12 and Pro17 appear at *j* = 1190.

Just after the formation of the hydrophobic core, as shown in Figure 3, the salt bridge between Asp9 and Arg16 is formed for the time step indices 1200 ≤ *j* ≤ 1250. According to the experimental studies [1, 6, 8–10], the salt bridge is essential for Trp-cage stability in solution. The distance of the salt bridge decreases from 9.9 Å at *j* = 1200 to 5.1 Å at *j* = 1250. At the same time (1200 ≤ *j* ≤ 1250), together with the salt-bridge formation, the radius of gyration increases from 7.1 Å to 7.4 Å (as shown in Figure 1), indicating the slight expansion of Trp-cage, but RMSD continuously decreases from 3.9 Å to 3.3 Å. It should be noted that the value of *R*_*g*_ = 7.4 Å at *j* = 1250 is the same as that of the native structure. It seems that a near-native intermediate [11] is formed in this stage.

After the formations of the hydrophobic core and the salt bridge (i.e., after *j* = 1250), there is no remarkable event, as shown in Figures 1 and 3. In particular, after *j* = 1250, RMSD decreases slowly and the numbers of native contacts and hydrogen bonds increase consistently, implying the slow adjustment processes of the near-native states into the native structure [17].

### 3.3. Principal Component Analysis

As a further analysis of the Trp-cage folding pathway, we use the method of principal component analysis (PCA) [36] that best describes the protein structural changes and is a mathematical method for analyzing correlations in large data sets. In usual applications, PCA can be used for dimensionality reduction in a data set while retaining the characteristics of the data set that contribute most to its variance. In the present application, PCA extracts the essential motions in the protein folding events through the ADMD simulation. We can easily validate the usefulness of the analysis by characterizing the percentage of the variance with a chosen set of principal components. To be a useful method in protein folding event analysis, one should provide the long-time protein folding dynamics before the PCA study. In this sense, the present ADMD pathway provides a good input data set. The reason for this is that the ADMD method is a double-ended formulation containing a global feature of the protein folding events.

Here, we define the covariance matrix *C* of the spatial fluctuation as

(8) $$\begin{array}{c} C_{ij}=\langle \left(x_{i}-\langle x_{i}\rangle \right)\left(x_{j}-\langle x_{j}\rangle \right)\rangle , \end{array}$$

where *x*_1_, *x*_2_, *x*_3_,…, *x*_3*N*_*α*__ are the Cartesian coordinates of the *N*_*α*_C^^*α*^^ atoms. The average 〈…〉 is over all structural frames from the ADMD trajectory (i.e., *P* + 1 = 2001). The matrix contains information on the spatial correlation between residue pairs.

The correlation matrix *C* provides information on the correlated fluctuations of C^^*α*^^ atoms in the folding process. To analyze the protein folding pathway, we compute the principal components, the 3*N*_*α*_ eigenvalues, and their corresponding eigenvectors from the correlation matrix. Upon diagonalization of the 3*N*_*α*_ × 3*N*_*α*_ correlation matrix, a set of eigenvalues and eigenvectors is obtained. Eigenvectors with large eigenvalues correspond to the directions of large conformational fluctuation in the pathway. It turns out that a large part of the molecule's fluctuations can be obtained in terms of only few PCA eigenvectors, corresponding to the eigenvectors with the largest eigenvalues. The eigenvalues of the correlation matrix are proportional to the average-squared fluctuations in the configurational space along the corresponding directions of the eigenvectors. This is helpful in analyzing the motions of flexible regions in proteins. The flexible regions will be expressed by large values of the variance of the Cartesian coordinates.

**Figure 5:**
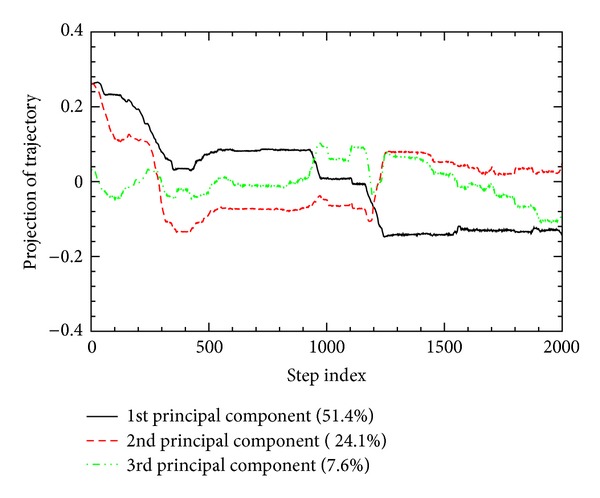
The folding trajectory is projected to the first three principal components, which are responsible for a total of 83.2% of the covariance matrix. For each principal component, the percentage of total variance is also shown.

The projections of the protein folding pathway onto the eigenvectors corresponding to the three largest eigenvalues of the correlation matrix, as a function of the time step index *j*, are shown in Figure 5. These principal components can serve to describe the protein folding events in terms of 83.2% of total fluctuations. As shown in Figure 5, the variations of the first three principal components reflect well the three major events around *j* ~ 300, 950, and 1200, described in the previous subsections. The first principal component (responsible for 51.4%) changes greatly around *j* ~ 300, 950, and 1200, following the variations in Figures 1 and 3. The second principal component (24.1%) quite varies around *j* ~ 300 and 1200, but not much around *j* ~ 950. The third principal component (7.6%) changes much around *j* ~ 950 and 1200, and gradually after *j* ~ 1300. This gradual change of the third principal component is concomitant with the slow adjustment process of a near-native state into the native structure [17], noticed in the variation of RMSD (Figure 1).

**Figure 6:**
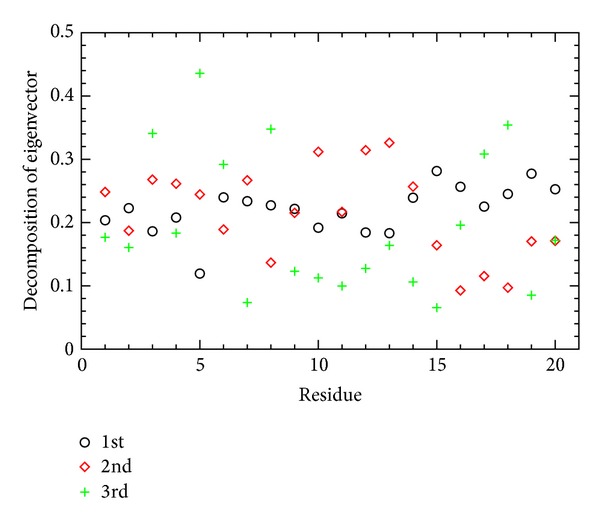
The C^*α*^ atom contribution to the first three principal components is shown as a function of residue index. The *y*-axis corresponds to the magnitude of three components (*x*, *y*, and *z*) contributing to the corresponding principal component. Higher values on the *y*-axis indicate more active contributions to the fluctuations.

Figure 6 shows how the atoms contribute to the principal components, measured by the C^^*α*^^ atomic fluctuations through the ADMD simulation. A notable contribution to the C^^*α*^^ atomic fluctuations during the protein folding process is identified once again through the PCA method. As far as the first principal component concerned, the C-terminal side is more flexible than the middle part of the protein. The second principal component is mainly derived from the middle part of the protein where a relatively inactive contribution is found in the first principal component.

**Figure 7:**
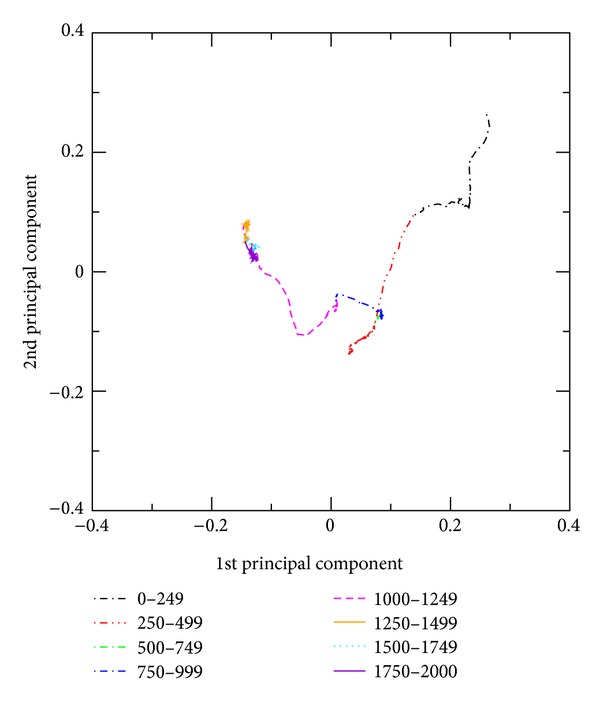
The Trp-cage trajectory is projected into the first two principal components. These two components account for over 75% of total variance, allowing most of the folding information to be plotted in two dimensions. Eight different ADMD step-index intervals are indicated with different colors.

The PCA projections of the protein folding pathway onto the plane characterized by the two principal components with largest variances are shown in Figure 7. The first two components account for over 75% of total variance, allowing most of the folding information to be plotted in two dimensions. The eight different segments of the two-dimensional version of the protein folding trajectory are presented in the figure. A relatively slow progress in the fluctuations is found at the step indices 500 ≤ *j* ≤ 749 where there is no noticeable change in *R*_*g*_, RMSD, potential energy, the numbers of native contacts and hydrogen bonds, and the distances from the side chain of Trp6 (see Figures 1 and 3.). It implies that the unfolded state formed after *j* ~ 300 is quite stable. On the other hand, the slow progresses for 1250 ≤ *j* ≤ 2000 correspond to the slow adjustment processes of the near-native states into the native state [17].

## 4. Conclusion

We have studied the dynamic folding pathway models of the 20-residue Trp-cage protein into the native structure at all-atom resolution by using ADMD and parallel computation with the AMBER force field and the GB/SA solvation potential. In ADMD simulations, the chain of conformations with dynamic information is obtained, connecting the initial conformation and the final native conformation of Trp-cage, by applying the least action principle. We have performed twenty independent ADMD simulations where initial pathways are prepared in a random fashion, producing variation in pathways. However, these twenty pathways show similar potential-energy fluctuation along their trajectories, and overall folding features independent of initial randomness have been considered. We have found that the radius of gyration, RMSD from the native structure, and potential energy are correlated with each other, along the time step index *j* ( = 0,1,…, 2000). Also, the numbers of native contacts and native hydrogen bonds are highly correlated, implying that the native structure of Trp-cage is achieved through the concurrent formations of native contacts and native hydrogen bonds.

In early stage (*j* ~ 300), an unfolded state appears with partially formed native contacts (~40%) and native hydrogen bonds (~30%). It includes two partially formed 3_10_ helices in the N-terminal and middle fragments. In the unfolded state, the side chain of Trp6 is in contact with the residues (Gly11 and Pro12) of the middle 3_10_ helix, consistent with the experimental studies [3, 5]. For the time step indices 300 ≤ *j* ≤ 900, there is no remarkable change, and the unfolded state is quite stable.

Around *j* ~ 950, the side chain of Tyr3 begins to be in contact with that of Trp6, together with the formation of the N-terminal *α*-helix. According to a recent experimental study [8], the contact between the side chains of Tyr3 and Trp6 is the key interaction in the folding processes. For the time step indices 1150 ≤ *j* ≤ 1200, the C-terminal polyproline II structure docks onto the Trp6 and Tyr3 rings of the partially formed N-terminal *α*-helix, resulting in the formation of the hydrophobic core of Trp-cage. Immediately, the salt bridge between Asp9 and Arg16 is formed and provides the stability for the hydrophobic core of Trp-cage. In this stage, a near-native intermediate [11] seems to be formed.

Furthermore, the method of principal component analysis has been used in the understanding of the Trp-cage folding processes. The first principal component (responsible for 51.4% of total fluctuations) changes greatly at *j* ~ 300, 950, and 1200, in excellent agreement with the variations in the other measures. The first two principal components account for over 75% of total variance, allowing most of the folding information to be plotted in two dimensions. This analysis indicates that the slow adjustment processes of the near-native states into the native structure are dominant in later stage (1250 ≤ *j* ≤ 2000).
